# Biomedical Processing of Polyhydroxyalkanoates

**DOI:** 10.3390/bioengineering6040108

**Published:** 2019-11-29

**Authors:** Dario Puppi, Gianni Pecorini, Federica Chiellini

**Affiliations:** Department of Chemistry and Industrial Chemistry, University of Pisa, UdR INSTM – Pisa, Via G. Moruzzi 13, 56124 Pisa, Italy; gianni.pecorini21@gmail.com

**Keywords:** polyhydroxyalkanoates processing, electrospinning, additive manufacturing, selective laser sintering, fused deposition modeling, computer-aided wet-spinning

## Abstract

The rapidly growing interest on polyhydroxyalkanoates (PHA) processing for biomedical purposes is justified by the unique combinations of characteristics of this class of polymers in terms of biocompatibility, biodegradability, processing properties, and mechanical behavior, as well as by their great potential for sustainable production. This article aims at overviewing the most exploited processing approaches employed in the biomedical area to fabricate devices and other medical products based on PHA for experimental and commercial applications. For this purpose, physical and processing properties of PHA are discussed in relationship to the requirements of conventionally-employed processing techniques (e.g., solvent casting and melt-spinning), as well as more advanced fabrication approaches (i.e., electrospinning and additive manufacturing). Key scientific investigations published in literature regarding different aspects involved in the processing of PHA homo- and copolymers, such as poly(3-hydroxybutyrate), poly(3-hydroxybutyrate-*co*-3-hydroxyvalerate), and poly(3-hydroxybutyrate-*co*-3-hydroxyhexanoate), are critically reviewed.

## 1. Introduction

The always increasing interest on polyhydroxyalkanoates (PHA) for biomedical applications stems from their well-ascertained biocompatibility and biodegradability in physiological environments [1]. In addition, their microbial synthesis by means of sustainable processes with potential for large-scale industrial production [2], together with a better processing versatility and superior mechanical properties in comparison with other polymers from natural resources, make PHA unique polymer candidates for advanced research and development approaches.

**Figure 1:**
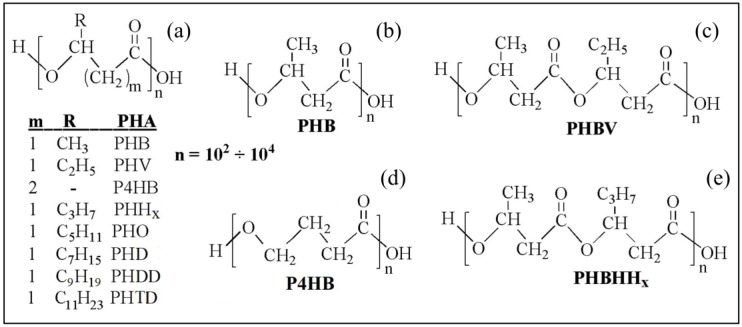
(**a**) General chemical structure of polyhydroxyalkanoates (PHA); chemical structure of (**b**) poly(3-hydroxybutyrate) (PHB), (**c**) poly(3-hydroxybutyrate-*co-*3-hydroxyvalerate) (PHBV), (**d**) poly(4-hydroxybutyrate) (P4HB), and (**e**) poly(3-hydroxybuyrate-*co-*3-hydroxyexanoate) (PHBHHx).

From a chemical point of view, PHA are aliphatic polyesters with a variable number of carbon atoms in the monomeric unit (Figure 1). They are generally classified as short-chain length (SCL)-PHA when they consist of monomers C3-C5 in length, and medium-chain length (MCL)-PHA when they consist of monomers C6–C14 in length [3]. SCL-PHA consist of monomeric units of 3-hydroxybutyrate (3HB), 4-hydroxybutyrate (4HB), or 3-hydroxyvalerate (HV). MCL-PHA consist of monomeric units of 3-hydroxyhexanoate (HHx), 3-hydroxyoctanoate (HO), 3-hydroxydecanoate (HD), 3-hydroxydodecanoate (HDD), 3-hydroxytetradecanoate (HTD), or even longer-chain comonomer units [4]. In general, SCL-PHA have high crystallinity degree and behave as a stiff and brittle material, while MCL-PHA have reduced crystallinity and increased flexibility showing elastomeric properties. The length of the pendant groups of the monomer units plays a key role in the resulting polymer physical properties, so that SCL-PHA copolymers with ethyl side groups can show elongation at break values varying in the range 5–50% depending in comonomers units ratio [5]. In addition, copolymers consisting of both SCL- and MCL-subunits can have properties between those of the two states. While most bacteria accumulate PHA granules of only one type, i.e., SCL or MCL, bacteria accumulating SCL-MCL-PHA copolymers were also isolated [6].

Poly(3-hydroxybutyrate) (PHB), which was the first discovered PHA in the 1920s [7], together with its copolymers poly(3-hydroxybutyrate-*co-*3-hydroxyvalerate) (PHBV), represent the most investigated microbial polyesters in the biomedical area thanks to the thermoplastic behavior, mechanical properties suitable for load-bearing applications, and versatile synthesis methods [8,9]. Novel microbial synthesis procedures have allowed the biomedical investigation of PHA with a wide range of molecular structures, in terms of molecular weight, length of alkyl side group, and ratio of comonomer units, as a means to develop materials with physical properties tailored to specific applications [10,11]. A widely investigated example in this context is represented by poly(3-hydroxybuyrate-*co-*3-hydroxyexanoate) (PHBHHx), which shows tunable elasticity by varying HHx percentage, exploitable for different applications, such as engineering tissues with much different stiffness (e.g., bone, cartilage, nerve, and blood vessel) [12]. Thanks to its low crystallinity, poly(4-hydroxybutyrate) (P4HB) has higher flexibility, ductility, and processing properties in comparison to PHB, which have been exploited to develop biomedical devices for soft tissue repair [13]. P4HB products currently on the clinical market include, among others, GalaFLEX®, a surgical mesh of knitted fibers [14], TephaFLEX® sutures, meshes, tubes, and thin films [15], MonoMax® sutures [16], Phantom Fiber™ sutures and BioFiber® Surgical Mesh [17].

PHA versatility in terms of processing approaches and conditions has allowed the investigation and application of a wide range of fabrication techniques relevant to biomedical research and industrial application. As it will be discussed in detail in the following sections, techniques based on different working principles have been successfully employed to process PHA, either in the form of a melt or a solution, and shape them at different scale length levels. Techniques with an old industrial history, such as melt spinning and blow extrusion, are currently employed to produce implantable medical devices made from PHA that are available on the market. Other approaches industrially employed in the case of commodity use polymers, such as solvent casting and injection molding, or under development for porous polymer structures fabrication, such as freeze drying and phase separation methods, have been also optimized for PHA processing. In this context, this review article is aimed at summarizing PHA processing properties in relationship to the requirements of the different techniques developed so far for their processing, as well as at critically overviewing key literature on this topic. Emphasis is dedicated to cutting edge advancement reported in literature on electrospinning and additive manufacturing application to PHA processing.

## 2. Physical and Processing Properties of PHA

As previously mentioned, the side group length significantly affects PHA crystallinity, mechanical behavior, as well as processing properties. PHB has a glass transition temperature (T_g_) ~0 °C, melting temperature (T_m_) ~180 °C, crystallinity degree in the range 60–80%, and degradation temperature (T_deg_) ~220 °C. The narrow window between T_m_ and T_deg_, typically requires the use of plasticizers for PHB melt processing in order to prevent polymer decomposition, which can occur at temperatures above 150 °C, as well as to enhance melt strength and elasticity [13,18]. By increasing the molar percentage of HV in copolymers, T_m_ can be decreased down to 130 °C and crystallinity to 35%, without marked effect on T_g_ and T_deg_, thus widening processing temperature window and enhancing melt processability. Analogously, in the case of PHBHHx, T_m_ can be decreased down to 54 °C and crystallinity to 15%. Values of Young modulus and elongation at break reported in literature for PHBV (0.5–3.5 GPa, 5–50%) and PHBHHx (0.1–0.5 GPa, 5–850%) are generally different than those reported for PHB (0.9–4.0 GPa, 5–20%). As a consequence of the absence of alkyl side groups along the macromolecular chain, P4HB has much lower T_g_, (~ −50°C), T_m_ (55–70 °C), and crystallinity (<40%) than PHB, resulting in enhanced melt processability, lower stiffness, and much larger elongation at break. These differences justify the successful application of different melt processing techniques for the fabrication of biomedical products currently available on the market, as discussed in the next section. Copolymers of 3HB and 4HB monomeric units, poly(3-hydroxybutyrate-*co*-4-hydroxybutyrate) (P3HB-*co*-4HB), show thermal and morphological parameters, as well as mechanical and processing properties, in between those of the two relevant homopolymers [19].

SCL-PHA are soluble only in a few organic solvents, including chloroform, dichloromethane, dimethyl formamide, tetrahydrofuran, and dioxane. In addition, their solubilization can require high temperatures or sonication to form a homogeneous solution at concentrations suitable for processing techniques commonly used in the biomedical field (e.g., solvent casting, phase separation, and electrospinning) [20]. This aspect is particularly significant in the case of PHB often resulting in suspensions rather than homogeneous solutions when mixed with organic solvents.

**Figure 2:**
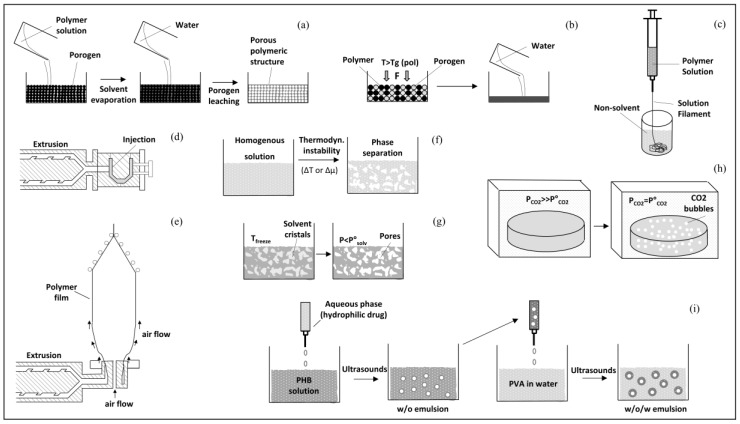
Schematic representation of techniques commonly employed for PHA processing in the biomedical area (modified from [23]). (**a**) Solvent casting-particle leaching process: a polymer solution is cast into a mold filled with porogen particles, the solvent is allowed to evaporate and the porogen is finally water-leached out; (**b**) melt molding-particulate leaching: a powder mixture of polymer and porogen is placed in a mold and heated above the polymer T_g_ while a pressure (F) is applied, the porogen is then water-leached out; (**c**) representative fiber spinning technique, i.e., wet-spinning: a polymeric solution is extruded directly into a coagulation bath leading to the formation of a continuous polymer fiber by non-solvent-induced phase inversion; (**d**) injection molding: a polymeric material is melt-extruded and injected into a mold; (**e**) film extrusion: a polymeric material is melt-extruded in the form of a tubular film by using a circular die and air pressure; (**f**) phase separation: a thermodynamic instability is established in a homogeneous polymer solution that separates into a polymer-rich and a polymer-poor phase; (**g**) freeze drying: a polymer solution is cooled down leading to the formation of solvent ice crystals, then a pressure lower than the equilibrium vapor pressure of the solvent (P_solv_) is applied; (**h**) gas foaming: a chemical or physical blowing agent is mixed with the polymeric material, typically during extrusion (in the case depicted in figure a polymeric sample is exposed to high pressure CO_2_ allowing saturation of the gas in the polymer and the subsequent gas pressure reduction causes the nucleation of CO_2_ bubbles); (**i**) double emulsion for particles preparation: an aqueous phase containing a drug is added to a polymer solution, a water-in-oil (w/o) emulsion is obtained through sonication and added to a second aqueous phase, to form a w/o/w emulsion, particles are then separated by centrifugation, after organic solvent evaporation under stirring, and possibly resuspended in an aqueous phase.

The theoretical and practical aspects of the most exploited processing techniques in the biomedical field, as well as of advanced fabrication approaches based on electrospinning [21] and additive manufacturing [22], are overviewed in the following section. Schematic representations of various techniques commonly employed for PHA biomedical processing are reported in Figure 2. They include techniques to fabricate 3D molded objects (e.g., injection molding), films (e.g., blow extrusion), continuous fibers (e.g., wet-spinning), nanoparticles (e.g., double emulsion), as well as a number of processing approaches to obtain a porous structure. The latter are due to a widespread interest raised within the scientific and clinical communities for the development of biodegradable scaffolds with a porous architecture tailored to tissue regeneration strategies. 

## 3. Biomedical Processing of PHA

**Figure 3:**
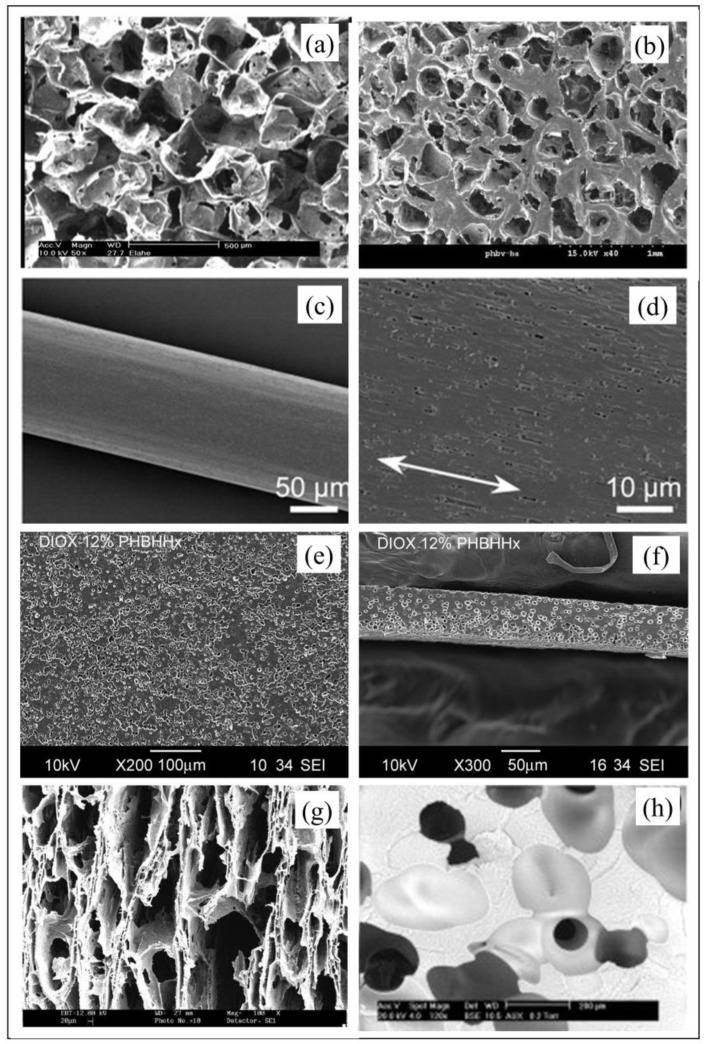
Scanning electron microscopy (SEM) analysis of PHA morphology after processing: (**a**) PHB porous structure by solvent casting/particulate leaching (scale bar 500 μm; reproduced from [24]); (**b**) PHBV/hydroxyapatite (HA) porous structure by melt molding/particulate leaching (scale bar 1 mm; reproduced from [25]); (**c**) low and (**d**) high magnification analysis of PHBV fiber fabricated by melt-spinning, and subjected to isothermal crystallization and 10 times one-step-drawing at room temperature (reproduced from [26]); (**e**) top view and (**f**) cross-section of PHBHHx film prepared by phase inversion of a 1,4 dioxane solution through immersion in water (reproduced from [27]); (**g**) PHBV scaffold by means of emulsion freezing/freeze drying technique (scale bar 20 μm; reproduced from [28]); (**h**) PHBV porous morphology obtained by gas foaming (scale bar 200 μm; reproduced from [29]).

Given the thermoplastic behavior and solubility in organic solvents of this class of polyesters, various approaches have been investigated for processing PHA into systems with different potential biomedical applications. Representative tailored processing strategies to obtain PHA-based constructs with a morphology engineered at different scale levels are described in the following, as seen in Figure 3. A particular focus is given to electrospinning and additive manufacturing, which represents the most advanced processing approaches with great potential for biomedical industry translation. Electrospinning is the technique of election for fabricating PHA nanofibers organized into 3D assemblies with structural features mimicking those of the native tissues’ extracellular matrix. This aspect together with other inherent advantages, including high surface to volume ratio of ultrafine fiber systems and processing versatility for drug-loading, make electrospinning of PHA suitable for a wide array of biomedical applications, as discussed more in depth in Section 3.1. Moreover, combining the sustainable production potential of PHA with the high technological level of additive manufacturing, in terms of reproducibility, automation degree, and control on composition and structure at different length scales, is inspiring a growing body of current literature that can have a tremendous impact on the biomedical industry.

**Solvent casting** represents one of the most straightforward approaches to process PHA into two-dimensional (2D) membranes. For instance, Basnett et al. [24] developed films based on poly(3-hydroxyoctanoate) (PHO) blended with PHB by dissolving the two polymers in chloroform at different weight ratios (80:20, 50:50 or 20:80) for a total concentration of 5% wt. The solutions were mixed by sonication, cast into a glass petri dish, and then air dried for one week to obtain films of 180–220 µm thickness. Drying in an atmosphere saturated with the solvent is often employed to achieve slow solvent evaporation and avoid internal stress formation. Combination of solvent casting with salt leaching is an effective means to obtain a PHA porous structure. As an example, Masaeli et al. [30] added NaCl particles (200–250 µm) to chloroform solutions of PHB and, after solvent evaporation and vacuum drying, submitted the solid to extensive water washing for five days. The resulting salt-leached membranes have a thickness of around 500 µm and a porosity of around 90% (Figure 3a). Advantages of this processing approach is the ease of fabrication, the possibility to vary the pore’s size over a large range, as well as to control pore size and porosity independently. The main limitation is that only small thicknesses can be achieved due to difficulty in removing salt particles along thick sections. In addition, membrane shape is given by the mold, and obtaining customized geometries requires designing and fabricating ad-hoc molds.

**Melt molding** can be alternatively employed to fabricate thin PHA membranes, possibly in combination with salt leaching for developing porous architectures. After filling a mold with polymer and porogen particles, the system is heated above polymer T_g_, while applying pressure to the powder. Once the polymer particles are fused together, the mold is removed and the porogen is leached out. As an example, PHBV/ hydroxyapatite (HA) powder (9:1 w/w) was mixed with NaCl particles (100~300 μm) at a 1:17 weight ratio and then cast in a mold at 180 °C [25]. After leaching out salt particles, through water washing, and drying it under a vacuum, PHBV scaffolds exhibited an interconnected, porous network with pore sizes ranging from several microns to around 400 μm (Figure 3b). This processing approach holds some advantages and disadvantages of solvent casting-based techniques, such as the independent control of pore shape and porosity, the ease of fabrication, the limited design freedom in terms of membrane shape and thickness. Moreover, while it avoids the use of organic solvents that can be harmful for biological systems, it requires high temperature processing with the related risks of thermal degradation and energy costs.

**Fiber spinning** techniques, i.e., melt-, dry-, and wet-spinning, were recently investigated to process PHBV into single fibers or tridimensional (3D) fibrous macroporous scaffolds. They involve the extrusion of a polymer as a melt, in the case of melt-spinning, or dissolved in a solvent and then extruded in air or directly into a coagulation bath, in the case of dry- or wet-spinning, respectively. Depending on the technique employed, the final applications, and other product requirements, the fibers are submitted to different post-processing treatments (e.g., drying, washing, and drawing) or assembled into 3D fibrous systems. In the case of melt spinning, different methods have been investigated to overcome PHA processing shortcomings, such as adhesion, high brittleness, and low melt strength, related to slow crystallization rate, large spherulite size, and secondary crystallization [31]. Blending with organic or inorganic particles acting as nucleating agents, graft copolymerization, or fiber stretching are effective means to control and optimize the crystal structure and crystallization behavior of PHBV. For instance, PHBV fibers with around 1 GPa tensile strength were prepared by quenching during melt spinning, followed by isothermal crystallization near the T_g_, and one-step-drawing at room temperature (Figure 3c) [26]. As previously mentioned, melt spinning is commercially employed for producing monofilaments or multifilaments made of P4HB (T_m_ ~60 °C) that are used as sutures or further processed using conventional textile processes, such as braiding, knitting, and weaving, to produce scaffolds and surgical meshes [14,15]. In particular, P4HB monofilament sutures show superior tensile strength characteristics than polydioxanone and polypropylene sutures. Wet-spinning technique has been investigated to overcome the aforementioned shortcomings related to thermal processing of PHA. For instance, Alagoz et al. [32] extruded a chloroform solution of PHBV into a coagulation bath of methanol. The fibers produced were kept in methanol overnight at −4 °C for solidification, then placed into a cylindrical Teflon mold, and dried in a vacuum oven. The resulting scaffolds had a diameter of 4 mm, height of 2 mm, interconnected porosity of 75%, average fiber diameter of 90 µm, and pore size of 250 µm.

**Injection molding and film extrusion** are also used to process PHA into 2D or 3D objects with potential application in the biomedical industry. These techniques involve processing the polymer in a screw extruder, pumping the melt through a die. The melt is either injected in a mold, or axially drawn and radially expanded in the form of a thin-walled tube to obtain a continuous film. Injection molding can be also combined with the particulate leaching strategy, or integrated with blowing agents that are blended with the raw polymer and activated upon heating to form a porous structure [33]. A range of melt extrusion grade formulations based on PHB or PHBV blended with additives, other polymers, and/or inorganic fillers, have been developed to enhance the material toughens processability, as well as to reduce costs. They are currently available on the market for applications other than medical ones. Examples are injection molding and film blowing grade PHA formulations approved for food contact that are marketed by Telles and TianAn [34]. Although the employment of these melt processing approaches to biomedical research is limited, the trademark TephaFLEX® by Tepha Inc. includes, besides the previously cited sutures produced by melt spinning, P4HB surgical tubes and films made by injection molding and blow extrusion, respectively [15].

**Phase separation** approaches are widely investigated for the preparation of porous PHA systems. They generally rely on establishing a thermodynamic instability in a polymer solution, through changes of physical conditions (e.g., temperature) or chemical composition (e.g., non-solvent addition), to induce a separation into two phases at different composition. Li et al. [35] obtained a nanofibrous network through phase separation by lowering the temperature of a PHB/chloroform/dioxane ternary mixture, with the resulting formation of a gel, which was then water-washed and freeze dried. This method was suitable also for the preparation of nanofibrous systems made of PHB blended with either PHBHHx or P4HB, whose tensile modulus, strength, and elongation at break could be modulated by varying the blend composition. Similarly, Tsujimoto et al. [36] obtained a microporous PHBHHx architecture by quenching a homogeneous polymer/DMSO solution that was prepared at 85 °C. Injectable formulations based on PHA can be prepared by dissolving the polymer in an organic solvent considered as not-toxic. This strategy is based on polymer film formation upon solution injection as a consequence of solvent dilution by the aqueous body fluids. Dai et al. [27] injected in the intra-abdominal position of rats formulations of PHBHHx dissolved in different solvents, i.e., N-methyl pyrrolidone, dimethylacetamide, 1,4-dioxane, dimethyl sulfoxide, and 1,4-butanolide. In particular, they found that PHBHHx films with a porous structure were formed when the solution came into contact with aqueous fluids because of a non-solvent-induced phase inversion process (Figure 3d). The wet-spinning methods described in this article also rely on a phase separation process induced by immersion into a polymer non-solvent [37]. 

**Freeze drying** is another processing approach investigated to fabricate porous PHA systems starting from a polymeric solution. As demonstrated by Sultana and Wang [28,38,39], porous scaffolds based on PHBV alone or in blends with poly(l-lactic acid) (PLLA), possibly loaded with HA, can be fabricated through an emulsion freezing/freeze drying process. In detail, the process involves adding an acetic acid aqueous phase to a polymer solution in order to obtain an emulsion that is then frozen and lyophilized. After sublimation, solvent (e.g., chloroform) and water phase crystals leave behind an anisotropic highly porous structure (Figure 3e). In the case of composite development, HA particles are added to the water phase before emulsion formation.

**Foaming** of PHA can be achieved by means of the employment of physical or chemical blowing agents, typically during melt extrusion. In the case of employment of physical agents, such as supercritical CO_2_, pressurized machinery with a more complex technology for gas pumping, screw extrusion, and pressure profile control is required [29]. Either exothermic (e.g., azodicarbonamide [40]) or endothermic chemical blowing agents (e.g., sodium bicarbonate and citric acid [41]) can be employed, the first ones releasing N_2_, the others releasing CO_2_. Epoxy-functionalized chain extenders and post-extrusion water-quenching have been proposed as effective means to enhance PHA foaming by increasing melt strength and controlling crystallization kinetics [42]. Although the great progress achieved on relevant processing aspects, foaming is not often used in the biomedical field since, despite the technological complexity, pores interconnectivity and surface porosity, which are a key requirement for most applications, are not easily obtained with this approach (Figure 3f).

**Formulation of nanoparticles,** microspheres, and microcapsules made from PHA has been widely investigated to develop biodegradable systems able to deliver pharmacologically active agents to a specific site of action, at the therapeutically optimal rate and dose regime [43]. Depending on drug hydrophilic/lipophilic behavior and the particle morphological requirements, different formulation methods can be employed, such as polymerization and nanoparticle formation in-situ, modified double emulsion-solvent evaporation, and oil-in-water emulsion-solvent evaporation [44]—as well as the dialysis method [45]. For instance, standard double-emulsion protocols for PHB nanoparticles preparation involve i) adding an aqueous phase, containing a drug and possibly an emulsifier, to an organic polymer solution under vigorous stirring or sonication ii) adding the obtained water-in-oil (w/o) emulsion to a second aqueous phase containing a hydrophilic polymer, e.g., poly(vinyl alcohol) (PVA), to form a w/o/w emulsion, iii) stirring until complete organic solvent evaporation, centrifugation, and resuspension in an aqueous phase. Folate-conjugated PHB nanoparticles loaded with an anti-cancer drug were recently prepared by following this method [46].

### 3.1. Electrospinning

**Figure 4:**
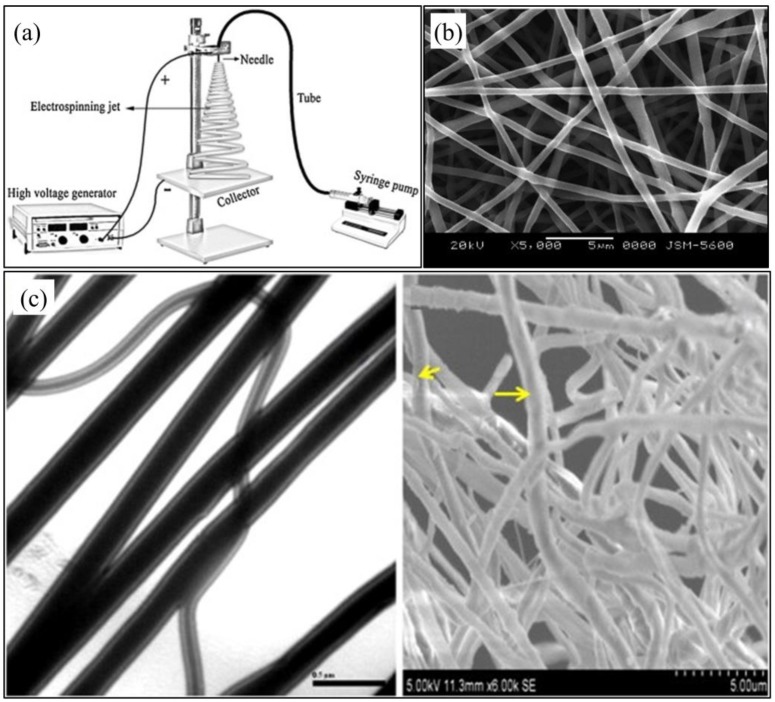
(**a**) Schematic representation of electrospinning set up (reproduced from [49]); (**b**) SEM micrograph of electrospun PHBHHx/ poly(d,l-lactic acid) (PDLLA) blend fiber mesh (reproduced from [50]); (**c**) TEM (left) and SEM (right) micrographs of PHB/gelatin core/sheath coaxial fibers (scale bars 0.5 μm and 5 μm, respectively; yellow arrows indicate the polymeric core/sheath structure; reproduced from [51]).

Electrospinning is the most employed technique for the production, on a lab and industrial scale, of polymeric nanofibers and nanofibrous meshes suitable for different applications, such as patches for tissue engineering and wound repair, nanostructured systems for drug release, filtration membranes, and protective and high-tech clothes [47]. This technique is based on an electrostatically-driven process that involves feeding a polymeric solution through a capillary into a high voltage electric field. The liquid drop is deformed, under the action of electrostatic forces and surface tension, assuming a shape similar to that of a cone. At a critical value of the applied voltage, a thin fluid jet is ejected at the apex of the cone and accelerated towards a grounded or oppositely-charged electrode, typically a flat metallic plate (Figure 4a). The stretching forces acting on the jet and the contemporary solvent evaporation, amplified by the violent whipping and splitting the jet undergoes during its travel, lead to the formation of fibers with a diameter in the range of a few micrometers down to tens of nanometers. The electrospun fibers can be collected in the form of nonwoven, yarn, 3D assemblies, and patterned structures, depending on the electrode/counter-electrode configuration [48].

The great interest by the biomedical science and engineering community on electrospinning is justified by its tremendous potential for the development of nanostructured systems designed for advanced tissue engineering and drug release applications. Indeed, electrospun nanofiber assemblies highly mimic the nanostructure of native extracellular matrix, thus providing cells with a 3D nanofibrous environment which allows them to better maintain their phenotypic shape and establish natural behavior patterns, in comparison to what observed in 2D cell culture and 3D macroporous architectures [52]. In addition, the simplicity and inexpensive nature of the fabrication setup making possible its scale up, and the high design freedom of fibers assembly architecture and composition, together with the versatility in the development of tailored drug-loading methods for functionalizing polymeric nanofibers with a wide variety of therapeutics, have led to a fast growing amount of literature published on electrospinning for drug release [53].

One of the first articles on electrospinning of PHA was published in 2006 and described an investigation of the relationship between processing parameters and electrospun fiber assembly morphology, as observed by means of SEM [54]. The study resulted in the development of a set of scaffolds made of PHB, PHBV, or their blend (75:25, 50:50, or 25:75 weight ratio) with average fiber diameter of few microns. High magnification SEM analysis showed that PHB/PHBV blend fibers had a rough surface, which was explained by the authors with a phase inversion process related to the rapid evaporation of chloroform, employed as a solvent. These scaffolds sustained the growth in vitro of mouse fibroblasts and human osteoblasts, at higher levels than analogous cast-films [55]. Electrospun PHB meshes were also recently shown to be a suitable substrate for human mesenchymal stem cells (MSCs) adhesion, proliferation, and differentiation [56,57]. As systematically investigated by Zhu et al. [58], the variation of PHBV concentration in the starting solution significantly influenced the morphology of the resulting fibers, likely because of an effect on chain entanglement during electrospinning [59]. They were able to change the electrospun structure by gradually increasing polymer concentration, from beaded to string-on-beads morphology, and then to uniform fibrous mesh, with a relevant increase of surface hydrophobicity, as a consequence of the increased roughness.

As reviewed by Sanhueza et al. [21], a current research trend is devoted to electrospinning PHA blended with other synthetic or natural polymers, with the aim of tuning the properties of the resulting fibers or endowing them with intrinsic bioactivity. Cheng et al. [50] processed PHBHHx and poly(d,l-lactic acid) (PDLLA) dissolved in chloroform mixed with dimethylformamide (DMF) (80/20 w/w) to increase the solution electrical conductivity. In particular, they showed that by increasing the PDLLA weight percentage from 25 to 50 or 75%, the tensile modulus was decreased and the elongation at break increased, while the biodegradation rate was higher, due to the more amorphous morphology of PDLLA in comparison to the semicrystalline nature of PHBHHx. The possibility of tuning PHBV meshes mechanical properties through blending with MCL-PHA was also recently shown. Indeed, electrospun meshes crystallinity, tensile strength, and modulus decreased, while the elongation at break increased, by blending PHBV with poly(3-hydroxyoctanoate-*co-*3-hydroxyhexanoate) (PHOHHx) (25% wt.), which is an amorphous MCL-PHA with elastomeric properties at room temperature. PHB blending with poly(l-lactide-*co-*ε-caprolactone) led to fiber diameter decreasing and hydrophobicity increasing, without any resulting effect on electrospun mesh mechanical properties [60]. An acetyl triethyl citrate/poly(vinyl acetate) blend was employed as a plasticizer and compatibilizer to improve the miscibility between PHB and poly(propylene carbonate) [61]. The resulting blend was electrospun into meshes with decreased crystallinity and T_m_ in comparison to PHB meshes. Nagiah et al. [51,62] investigated the modulation of the properties of PHB/gelatin membranes designed for skin tissue engineering, by adopting different electrospinning strategies. Indeed, by simultaneously electrospinning the two polymers with two separated syringes, processing a blend of the two polymers, or employing a coaxial electrospinning approach, they developed membranes composed by single gelatin fibers and PHB fibers, PHB/gelatin blend fibers, or biphasic fibers composed by a PHB core and a gelatin sheath, respectively (Figure 4c). The different fibers’ composition and architecture resulted in significant differences in mechanical properties, wettability, and proliferation of human dermal fibroblasts and keratinocytes cultured in vitro on the membranes. Zhinjiang et al. [63] demonstrated that a variation of PHB/cellulose ratio in a starting chloroform/DMF mixture significantly affected the biodegradation rate, as well as the wettability and mechanical properties of the resulting electrospun blend nanofibers. Blend fibers made of PHB/chitosan blends with different weight ratio were also electrospun by using trifluoroacetic acid, as a common solvent [64]. The addition of chitosan resulted in increased wettability and biodegradation rate, as well as decreased tensile strength. The possibility of tuning in a wide range of the tensile strength and elongation at break of zein/P3HB-*co-*4HB blend meshes by electrospinning was also recently shown by varying the weight ratio between the two polymers. Different chemical modification strategies have been also adopted to improve cell adhesion onto electrospun PHA meshes, e.g., epoxy functionalization [65] and polysaccharide-grafting [66,67]. Other PHA fibers functionalization approaches include combination with antibacterial particles (e.g., silver [68] and zinc oxide [69] nanoparticles) or electrosprayed osteoconductive ceramics (e.g., HA nanoparticles [70]), as well as grafting with carbon nanotubes mechanical-reinforcing fillers [71].

**Figure 5:**
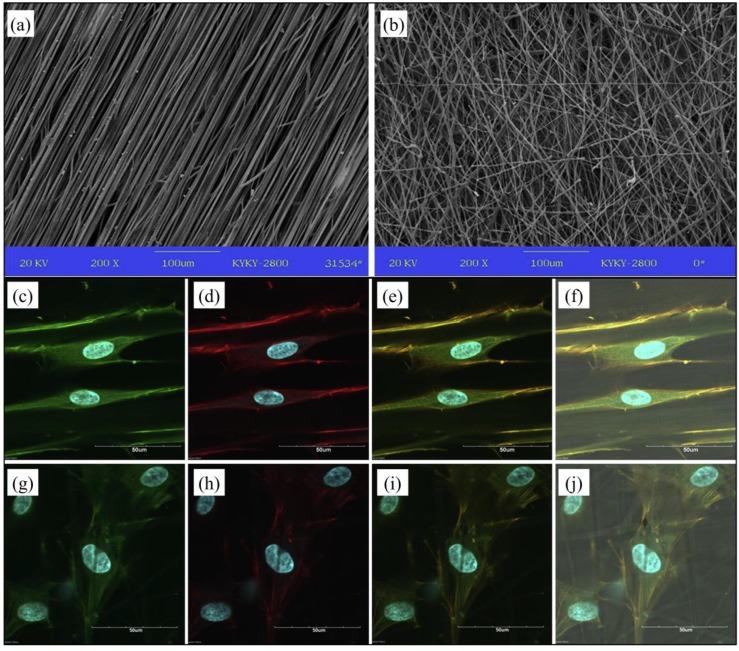
SEM micrographs of electrospun PHBHHx meshes composed by (**a**) aligned fibers or (**b**) randomly-oriented fibers (scale bar: 100 μm); confocal laser scanning microscopy images of MSCs cultured for three days on PHBHHx meshes composed by (**c**–**f**) aligned fibers or (**g**–**j**) randomly-oriented fibers (adhesion complexes (vinculin) in green, actin in red and nuclei in blue; scale bar in (**a**) and (**b**) 100 μm, scale bar in (**c**–**j**) 50 μm; reproduced from [75]).

A large body of literature has been dedicated to investigate and modulate electrospun PHA fibers organization and topography, as a means to control cell behavior and mesh mechanical properties. Yiu et al. [72] carried out a significant comparative study on the influence of topographic morphology of PHBHHx membranes fabricated by compression-molding, solvent-casting or electrospinning, on human MSCs adhesion, proliferation, and differentiation in vitro. Differently to what was observed in the two other kinds of membrane, MSCs showed a specific orientation on the electrospun fibrous meshes, exploitable for guided tissue regeneration and co-culturing of cells with orientation specificity (e.g., nerve, muscle and ligament cells). Aligned PHBV fibers systems can be fabricated by employing a rotating cylinder as fibers collector and auxiliary electrode [73]. Various studies have shown that fibers’ alignment can significantly influence physical-chemical, mechanical, and biological properties of the resulting membrane. For instance, an article reported enhanced wettability for PHBV aligned fibers in comparison to PHBV randomly-oriented fibers [74]. In addition, tensile testing revealed that the aligned PHBV fibers membranes were stronger in the longitudinal direction, but weaker in the transverse direction, in comparison to non-woven PHBV meshes showing instead an isotropic behavior. Fibers alignment resulted also in a different morphology of human osteosarcoma SaOS-2 cells that elongated when cultured in vitro. Similarly, Wang et al. [75] observed higher tensile modulus and strength, as well as increased MSCs elongation and differentiation, when electrospun PHBHHx fibers were aligned along their axes (Figure 5). The relationship between fibers’ alignment and mechanical properties was further investigated by a recent study reporting on electrospinning of PHB, P3HB-*co-*4HB, PHBV, and PHBHHx [76]. In all cases, fibers’ alignment resulted in enhanced tensile mechanical properties with an overall effect on surface properties. The employment of a rotating fiber collector has been also widely investigated for the production of non-woven meshes with a tubular geometry investigated, as suitable nerve conduits [77] or blood vessel scaffolds [78].

### 3.2. Additive Manufacturing

**Figure 6:**
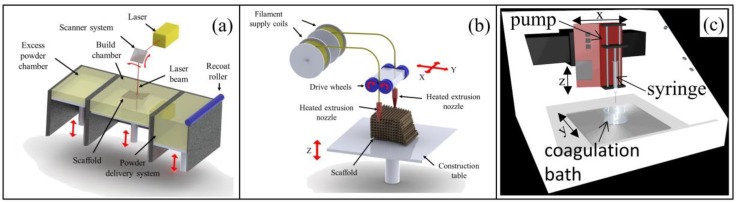
Schematic representation of additive manufacturing techniques applied to PHA processing: (**a**) selective laser sintering (SLS) and (**b**) fused deposition modeling (FDM) (reproduced from [80]); (**c**) computer-aided wet-spinning (CAWS) (reproduced from [86]).

As described in this section, various additive manufacturing approaches have been successfully applied to process different PHA, mainly into 3D porous scaffolds (Figure 6). Additive manufacturing was defined by ASTM as “the process of joining materials to make objects from 3D model data, usually layer upon layer” [79]. Additive manufacturing techniques are based on a computer-controlled design and fabrication process involving a sequential delivery of materials and/or energy to build up 3D layered objects. The geometrical and dimensional details, as well as other product specifications, such as density and composition gradients, are defined in a digital file which is then converted into a numerical control programming language, which specifies the motion of automated manufacturing tools. This approach enables advanced control over composition, shape, and dimensions of the object, in terms of design freedom and resolution. An advantage of additive manufacturing peculiar to the biomedical field is the possibility of deriving the 3D model data from medical imaging techniques commonly used for diagnostic purposes, such as computer tomography and magnetic resonance imaging. In this way, the anatomical features of biological tissues and organs can be reproduced through the fabrication process [80].

The various additive manufacturing techniques developed so far enable the processing of a wide range of materials by applying different approaches. Indeed, laser-based techniques are based on directing a beam or projection of light either to a photosensitive resin that is selectively photopolymerized, like in the case of stereolithography [81], or to a powder bed that is selectively sintered or fused, in the case of selective laser sintering (SLS) [82]. In the case of binder jetting, also referred to as 3D printing, a liquid binder, typically a polymer solvent, is deposited on a powder bed, which is selectively dissolved and fused upon solvent evaporation [83]. In extrusion-based techniques, a polymer in the form of a melt [84] or a solution/suspension [85] is extruded under controlled environmental conditions and selectively deposited onto a building stage. Fused deposition modeling (FDM) is a widely investigated example of melt-extrusion technique involving the extrusion and controlled deposition of a polymeric filament at a temperature above its T_g_. Computer-aided wet-spinning (CAWS) involves the controlled extrusion and deposition of a polymeric solution or suspension directly into a coagulation bath to achieve polymer solidification through a non-solvent-induced phase-inversion process. 

**Figure 7:**
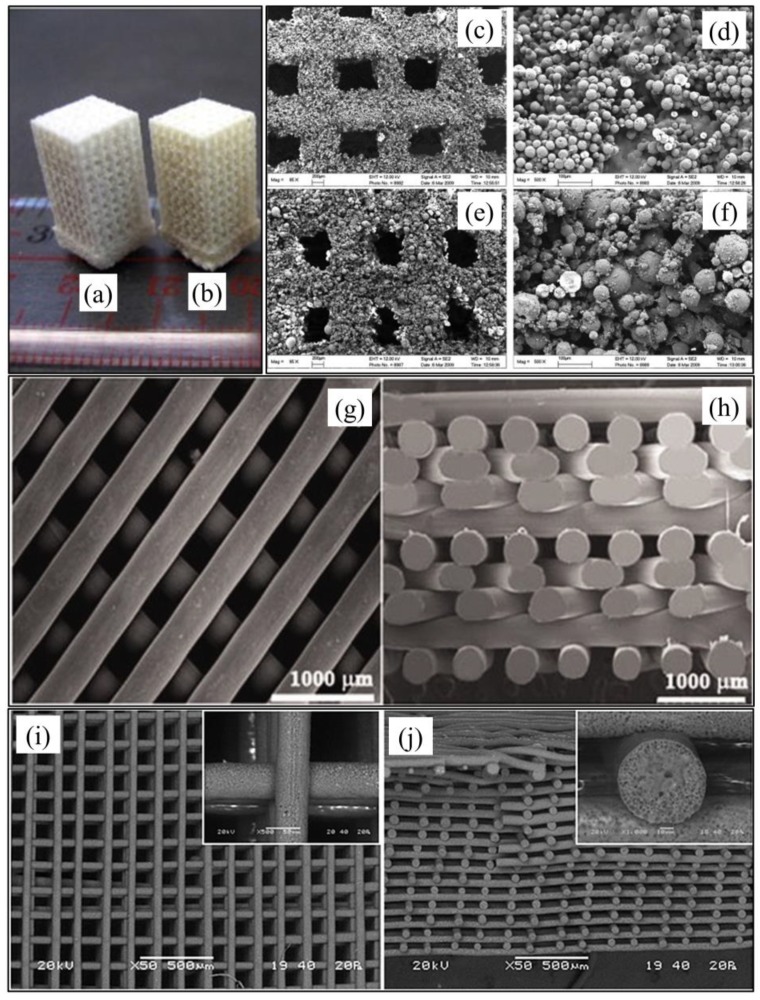
Additive manufactured PHA scaffolds: photograph of (**a**) PHBV and (**b**) CaP-loaded PHBV scaffolds by SLS, and relevant SEM micrographs of (**c**,**d**) PHBV and (e, f) CaP-loaded PHBV scaffolds (scale bars in (**c**) and (**e**) 200 µm, in (**d**) and (**f**) 100 µm; reproduced from [90]); SEM micrographs of (**g**) top view and (**h**) cross-section PHBV/PCL scaffolds by FDM (reproduced from [93]); SEM micrographs of (**i**) top-view and (**j**) cross-section of PHBHHx scaffolds by CAWS (inserts are high magnification micrographs, reproduced from [94]).

**Selective laser sintering (SLS)** was the first reported additive manufacturing technique to be employed for PHA processing (Figure 6a). In particular, different articles described the fabrication of 2D and 3D constructs with a predefined shape and interconnected porous architecture, through automated sintering of PHB particles, without any significant change in polymer chemical composition and thermal properties [87,88,89]. Duan et al. [90,91,92] published a few articles showing that PHBV scaffolds loaded with calcium phosphate (Ca–P) nanoparticles could be fabricated by processing nanocomposite microspheres previously prepared by means of an emulsion-solvent evaporation method (Figure 7a). They also showed that the scaffolds could be functionalized with the recombinant human bone morphogenetic protein-2 growth factor that was bound to a heparin coating applied on the surface. Although, this set of PHA scaffolds showed high fidelity on the macroscopic scale to the virtual model geometrical features, a low topographical resolution, was observed under SEM analysis (Figure 7c–f). This morphological feature, distinctive of SLS, is a consequence of an incomplete coalescence of polymeric particles during sintering due to the low laser energy employed to prevent thermal decomposition.

**Fused deposition modeling (FDM)** feasibility for processing PHA and exploiting their thermoplastic behavior has been assessed by a number of research activities (Figure 6b). However, the employment of FDM is still delayed in comparison to what achieved with other aliphatic polyesters, (e.g., PLLA), as a consequence of the limited thermal stability of PHA as well as their low melt elasticity and strength. For this reason, blending with other polymers or plasticizers is needed for successful PHA processing by FDM. Kosorn et al. [93] developed scaffolds made of poly(ε-caprolactone) (PCL)/PHBV blends with different weight ratios between the two polymers (75:25, 50:50, and 25:75) (Figure 7g,h). They demonstrated that the synergistic effect of increase in PHBV ratio and surface low-pressure plasma treatment significantly increased the proliferation and chondrogenic differentiation of porcine chondrocytes cultured in vitro in combination with the scaffolds. Blending with commercial monomeric plasticizers based on esters of citric acid (Citroflex®) was recently shown to be an effective means for FDM processing of PHB/PDLLA/plasticizer blends (60/25/15 wt.) into dog bone shape samples for tensile test [95]. Selection of the optimal plasticizer allowed a remarkable increase in elongation at break (from 5 to 187%) as well as a reduction of warping effects in the printed part upon solidification. The development of filaments made of maleic anhydride-grafted PHA loaded with palm fibers [96], wood flour [97], or multi-walled carbon nanotubes [98] was also recently reported in literature.

**Computer-aided wet-spinning (CAWS)** represents a successful example of hybrid additive manufacturing technique applied for the processing of PHA. CAWS approach involves the extrusion of a chloroform or tetrahydrofuran suspension of PHBHHx directly into a non-solvent bath (e.g., ethanol) to fabricate a device with a layer-layer process (Figure 6c). Under optimal conditions, the phase inversion process governing polymer solidification leads to the formation of a microporosity in the polymeric matrix integrated with the macroporous network created by the controlled deposition process (Figure 7i,j) [85,99]. PHBHHx scaffolds with different shape and porous architecture, resulting in varied mechanical response, can be fabricated by changing the design model and the processing conditions [94]. Indeed, anatomical PHBHHx scaffolds with the shape and dimensions of a critical size segment of a New Zealand rabbit’s radius model, and endowed with a longitudinal macrochannel for optimal bone regeneration conditions, were recently developed [100]. This kind of PHBHHx scaffolds, possibly in the form of a blend with PCL [101], were demonstrated to sustain the in vitro adhesion and proliferation of MC3T3-E1 murine preosteoblast cells. CAWS approach was also recently implemented with a rotating mandrel, as a fiber collector during polymer coagulation, to fabricate small-caliber biodegradable stents made of either PHBHHx or PCL [102]. Tubular constructs with different porous architectures were developed by controlling the synchronized motion of the deposition needle and the rotating mandrel.

## 4. Conclusions and Future Perspectives

Thanks to their thermoplastic behavior and suitable rheological properties when dissolved or suspended in proper organic solvents, processing of PHA has been investigated by adopting a number of tailored technological approaches. Indeed, the wide processing versatility given by the great variety, in terms of macromolecular structure and morphology, offered by PHA, makes this class of biodegradable polymers one of the most investigated in the biomedical field, especially when a load-bearing role is required. These aspects, together with the promising perspectives for PHA sustainable development [103], are propelling a fast growing research on relevant processing approaches to specific biomedical requirements. Both techniques with a long-history of industrial use, such as melt-spinning and foaming, and emerging techniques currently in the phase of being industrially-implemented, i.e., electrospinning and additive manufacturing, are applied for biomedical processing of PHA. The next frontier could be represented by the combination of different processing approaches to integrate in a single PHA device the high resolution down to the micro/nanoscale given by techniques like electrospinning and electrospraying, with the advanced control at the macroscale guaranteed by automated additive manufacturing approaches.
